# In Vitro Comparison of Microleakage of Two Materials Used as Pit and Fissure Sealants

**DOI:** 10.5681/joddd.2011.019

**Published:** 2011-09-05

**Authors:** Zahra Bahrololoomi, Aliasghar Soleymani, Zahra Heydari

**Affiliations:** ^1^Associate Professor, Department of Pedodontics, Faculty of Dentistry, Shahid Sadoughi University of Medical Sciences, Yazd, Iran; ^2^Dentist, Private Practice, Yazd, Iran

**Keywords:** Microleakage, flowable composite, fissure sealant

## Abstract

**Background and aims:**

Marginal seal of the material is extremely important in fissure sealant therapy. The aim of this study was to investigate microleakage of flowable composite resins and conventional fissure sealants with or without dentin bonding agent.

**Materials and methods:**

The occlusal surface of 60 intact extracted human premolars, divided into four groups, were cleaned with pumice/slurry, etched with 37% phosphoric acid for 15 seconds, rinsed and dried. Groups were treated differ-ently: Excite bonding agent followed by Helioseal F fissure sealant in group1; Helioseal F alone in group 2; Excite bonding agent followed by Tetric Flow in group 3; and Tetric Flow alone in group 4. Light-curing was done after each application. After thermocycling, the whole surface of each specimen was coated with nail varnish except for one millimeter around the fissure sealant. The teeth were immersed in 2% basic fuchsin for 24 hours and then sectioned buccolingually. The sections were analyzed for leakage under a stereomicroscope. Data was analyzed by Kruskal-Wallis and Mann-Whitney tests at asignificance level of P < 0.05.

**Results:**

There were no statistically significant differencesbetween the study groups in terms of the mean microleakage scores (P > 0.05), except for groups 2 and 4 (P = 0.002) and groups 3 and 4 (P = 0.033).

**Conclusion:**

Use of a flowable composite with bonding agent is a good alternative for sealing pits and fissures; however, further in vitro and in vivo studies are necessary.

## Introduction

In spite of various prevention methods, dental caries is still highly prevalent all over the world. Fluoridation of drinking water has become one of the most important methods for control of caries in smooth and proximal surfaces.^1^ Sealant placement is considered an effective treatment modality for prevention of caries in occlusal pits and fissures.^2^ Nevertheless, the preventive benefits of this treatment relies directly upon the ability of the resin sealant to thoroughly fill pits and fissures and/or morphological surface defects and remain completely intact and bonded to enamel for a life time.^3 , 4^

Adhesive agents have been used as mediating agents between the enamel surfaces and sealants. These materials have been advocated because of their low viscosity properties, which supposedly increase penetrability into occlusal pits and fissures.^5^

Several materials have shown good results when used as sealants for pits and fissures, and flowable composite resin is one of these materials.^2^

The applicability of flowable restorative systems in dentistry has increased, mainly because of their beneficial properties which include low viscosity, low modulus of elasticity and easy handling. Flowable composite materials have better abrasion resistance and, thus, provide a better retention than a conventional unfilled resin.^6 - 8^

However, some studies have indicated that increasing viscosity compromises flowable composite penetration into the etched enamel surface, thus, affecting sealing and retention.^2^

The marginal sealing ability of a sealing material is extremely important for success of treatment. Improper sealing can lead to marginal leakage, resulting in progression of caries underneath the restoration. In vitro microleakage studies can predict the marginal integrity of restorative materials.^9 , 10^

However, there appears to be few research studies comparing microleakage of such materials with that of conventional sealants. Therefore, this study evaluated whether a fissure sealant with a higher percentage of filler content would have less microleakage than a conventional pit and fissure sealant. The purpose of this study was to evaluate microleakage of a conventional resin-based sealant and a flowable composite resin.

## Materials and Methods

Sixty intact premolars extracted for orthodontic reasons were included in the study. The teeth were free of cracks, caries and restorations. Periodontal curettes were used for removal of remnants of soft tissues. The teeth were then stored in distilled water.^8 , 11 , 20^ Prior to the study, the occlusal surfaces of the teeth were cleansed with water/pumice slurry using brushes at low speed. The specimens were randomly divided into four groups (n = 15).

Initially, the specimens were gently air-dried. The enamel was acid-etched using 37% phosphoric acid (Ivoclar Vivadent, Liechtenstein) for 15 seconds. Air and water sprays were used for 10 seconds to completely rinse the acid and dry the teeth. In group 1, Excite bonding agent (Ivoclar Vivadent, Liechtenstein) was applied on the fissure for 15 seconds using a microbrush. Air spray was used for 5 seconds to evaporate the solvent. A halogen light-curing unit (Arialux; Apadana tak, Tehran, Iran) was used for 20 seconds to cure (500 mW/cm ^2 ^) the bonding agent. After placing the bonding agent, Helioseal F (Ivoclar Vivadent, Liechtenstein) was placed on the fissure and left there for 15 seconds. The sealant was then cured for 20 seconds according to manufacturer's instructions. In group 3, after application of Excite bonding agent, Tetric Flow (Ivoclar Vivadent, Liechtenstein) was applied on the fissures and light-cured for 20 seconds. The applied material and the experimental conditions in groups 2 and 4 were the same as those in groups 1 and 3, except that Excite bonding agent was not applied in the two latter groups. The teeth were thermocycled 500 times at 5 ± 2 / 55 ± 2°C. The surfaces of the specimens were coated with two layers of nail varnish except for one millimeter around the sealant. The specimens were stored in 2% fuchsin solution for 24 hours, rinsed and then sectioned buccolingually and longitudinally to assess dye penetration under ×25 magnification of a stereomicroscope (Sten SV 11-Zeiss, Germany).

A ranked scale was used to score dye penetration: (0) no dye penetration; (1) dye penetration limited to the outer half of the sealant; (2) leakage up to the inner half of the sealant; (3) dye penetration extending to the underlying fissure.

Data was analyzed by Kruskal-Wallis and Mann-Whitney tests at a significance level of P < 0.05.

## Results

**Figure 1:**
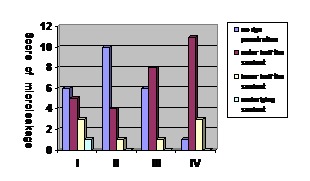
Comparative illustration of the groups. Group I: Excite bonding agent followed by Helioseal F fissure sealant; Group II: Helioseal F alone; Group III: Excite bonding agent followed by Tetric Flow; Group IV: Tetric Flow alone.

The present study aimed to comparatively assess the microleakage of teeth sealed with Tetric Flow and Helioseal F with and without the application of Excite bonding agent. Table 1 summarizes and Figure 1 illustrates the results of the present study.

**Table 1:** Dye penetration score for all the groups

	Dye penetration scores				
Group	0	1	2	3	Mean ± SD
1	6	5	3	1	0.93 ± 0.96
2	10	4	1	0	0.40 ± 0.63
3	6	8	1	0	0.66 ± 0.61
4	1	11	3	0	1.1 ± 0.51

Kruskal-Wallis test P value = 0.022 Group1: Excite bonding agent followed by Helioseal F fissure sealant; Group 2: Helioseal F alone; Group 3: Excite bonding agent followed by Tetric Flow; Group 4: Tetric Flow alone.

The mean microleakage score in group 4 was higher as compared to those of the other groups. However, there were no statistically significant differences between the study groups in terms of the mean microleakage scores (P > 0.05), except for groups 2 and 4 (P = 0.002) and groups 3 and 4 (P = 0.033) (Table 2).

**Table 2:** Comparison between groups using Man-Whitney test

Groups	P value
1 and 2	0.099
3 and 1	0.53
4 and 1	0.327
3 and 2	0.19
4 and 2	0.002
4 and 3	0.033

Group1: Excite bonding agent followed by Helioseal F fissure sealant; Group 2: Helioseal F alone; Group 3: Excite bonding agent followed by Tetric Flow; Group 4: Tetric Flow alone.

## Discussion

The present study aimed to comparatively assess microleakage of teeth sealed with Tetric flow and Helioseal F with and without the application of Excite bonding agent.

Fissure sealant therapy was introduced as a method to prevent occlusal caries more than 30 years ago. Since then, fissure sealant application has increased steadily and its effectiveness has been proven in many studies.^1 - 14^ Meticulous application procedures have resulted in high retention rates and high in vitro bond strengths.^1^

Sealant retention and integrity of the enamel–sealant interface determine, to a great extent, the caries reduction ability and effectiveness of a resin-filled fissure sealant.^1^

In vitro microleakage tests carried out with dye penetration technique are considered to be stricter than those performed in the oral cavity.^1^ This is most likely due to several factors such as the dye being more easily diffused than bacteria and their by-products and the fact that buildup of proteins in the marginal opening/gap may improve the seal. On this basis, they are likely to respond even better on a clinical level.^1^

The use of pumice prophylaxis followed by acid etching was chosen in this study because it is adopted by most dentists for application of sealants and is also recommended by the manufacturer.^19^

In this study, all the materials were applied without enameloplasty in order to observe the behavior of these materials without removal of tooth substance.

In an attempt to improve the retention of sealants and decrease microleakage, especially in conditions with unsatisfactory control of moisture, the use of adhesive systems with fissure sealant has been proposed.^20^ Therefore, in this study, the association of an adhesive system, Excite, with a flowable composite resin, Helioseal F, was chosen.

The findings of this study indicated that there were no statistically significant differences (P > 0.05) between the materials except in groups 2 and 4 and groups 3 and 4. Therefore, it is recommended that Tetric flow should not be used without a dentin adhesive.

On the basis of the results of the study and considering the importance of saving time in dental management of children, application of a fissure sealant is better than application of a sealant composite.

The superior results of Helioseal F seem to be related to its higher flowability rate, which is consistent with the results of previous studies that demonstrated sealant composites were similar to other conventional sealants and even showed more microleakage.^21 , 22^

Comparing classical sealants, flowable composite and flowable compomers has revealed that classical sealants show significantly lower microleakage than both flowable composite and flowable compomers.^8^ The reasons for greater microleakage are the nature of shrinkage that might affect the quality of sealing and also the use of bonding systems in combination with flowable composite, which might be another crucial step toward influencing the bond quality of sealants, thus affecting microleakage. High-modulus composites generally produce high shrinkage stresses during polymerization.

However, according to the results of a previous study, Tetric Flow sealant resin is superior in sealing deep caries-free fissures.^23^ The study results suggested the use of Helioseal F in special clinical situations with high caries risk and for instant fissure preservation in challenging situations for isolation of the tooth. Dental sealing in such conditions is temporary and they should be changed after clinical evaluation has been completed.^23^

Some authors have reported that use of a dentin bonding agent under sealants significantly decreases microleakage.^24 , 25^ These results were not confirmed by this study. Comparison of groups 1 and 2 in the present study showed that application of dentin adhesive is associated with less microleakage but is not significant. These results are similar to the findings of studies by Sirinvasan et al^26^ and Dukić and Glavina.^22^ However, Sirinvasan et al^26^ reported that the use of a bonding agent results in microleakage similar to that in samples restored without a bonding agent.

One of the limitations of this study was the fact that our study was an in vitro evaluation and moisture control was easy to achieve. These facts might explain the statistically similar behavior of the tested materials. Therefore, we recommend that similar studies with saliva contamination be performed. Further studies with other flowable restoration systems and different preparations must be carried out. In addition, in vivo investigations are necessary. In this context, parameters such as long-term retention and shear bond strength of flowable composite resin sealing must be considered.
